# Anthropometric formulas versus intracavitary ECG for optimal tip position of central venous catheters

**DOI:** 10.1186/cc13316

**Published:** 2014-03-17

**Authors:** MS Vallecoccia, M Biancone, F Cavallaro, D Settanni, C Marano, MG Annetta, M Pittiruti, M Antonelli

**Affiliations:** 1 Catholic University, Rome, Italy

## Introduction

Peres [1] and more recently Lum [2] developed some anthropometric formulas to correlate patient's height (H) and ideal length of central venous catheter (CVC) in order to identify optimal tip position. The aim of this study is to compare the reliability of the anthropometric formulas with the method based on intracavitary ECG.

## Methods

We enrolled patients admitted to our ICU candidate to elective CVC insertion, who had a detectable P wave on surface ECG. Intracavitary ECG was used to identify the optimum tip location since a maximal P wave indicates the cavo-atrial junction [3]. Post-insertion chest X-ray (CXR) was performed in all patients to verify the tip position. Assuming that the cavo-atrial junction is about 3 cm from the carina [4], the tip position was considered correct between 1 and 5 cm from the carina (between the lower 1/3 of the superior vena cava and the upper 1/3 of the right atrium). For each patient we retrospectively evaluated whether the catheter length calculated with Lum's and Peres' formulas on an estimated height would have been acceptable.

## Results

Sixty-five CVCs were placed: 51 in the right internal jugular vein (IJV), 14 in the left IJV. The mean catheter length by intracavitary ECG was significantly deeper than predicted by Lum's formulas (18.2 ± 1.9 vs. 16.7 ± 1.7 cm, *P *< 0.001) and not different from Peres' formulas (18.2 ± 1.9 vs. 18.2 ± 1.7 cm, *P = *0.8).

At post-procedural CXR, 88% of the tips were in the target zone. In three cases (5%) the catheter went in the wrong direction but was immediately corrected during the procedure since the intracavitary P wave did not change its amplitude. Compared with the intracavitary ECG, the incidence of malposition would have been significantly higher with Lum's formulas (48% vs. 12%, *P *< 0.001) and Peres' formulas (51% vs. 12%, *P *< 0.001).

## Conclusion

The intracavitary ECG was associated with a lower incidence of tip malposition than Peres' and Lum's formulas. It is also the only technique allowing one to immediately correct a primary malposition during catheter insertion.
